# Effects of cold plasma generated ozone on development of *Galleria mellonella* induced alterations in hemolymph protein and biochemistry of beeswax

**DOI:** 10.1038/s41598-026-36802-w

**Published:** 2026-02-10

**Authors:** Abeer O. Abotaleb, Hend H. A. Salem, Lina A. Abou El-Khashab, Eman F. Ebian, K. H. Metwaly

**Affiliations:** 1 Stord Product Pest Department, Plant Protection Research Institute, Agriculture Research Center, Giza, Egypt; 2 Zoology & Entomology Department, Faculty of Science, Al-Azhar University, Cairo, Egypt; 3 Center of Plasma Technology, Al-Azhar University, Cairo, 11884 Egypt

**Keywords:** Control, Cold plasma, Gas, Moth, Ozone, Wax, Entomology, Physics, Plasma physics

## Abstract

Greater wax moth *Galleria mellonella* is a serious pest in apiculture, invading honeybee colonies and inflicting widespread damage. Ozone gas generated by the non-thermal atmospheric pressure plasma produced with a dielectric barrier discharge (DBD) reactor is an attractive solution for insect control. The effect of ozone gas generated by cold plasma on the egg, larva, and pupae stages of *G. mellonella* was investigated by using two concentrations, 400 and 800 ppmv, at different exposure times of 5, 10, 20, 40, 60, and 80 min. The mortality rate of the larvae increased as the exposure time lengthened, while a decrease in adult survivorship was observed after exposure. Complete suppression of the survived larval instar percentage and pupation percentage was noted after 7 days of exposure to 40 and 20 min at 400 and 800 ppmv of ozone, respectively, compared to the control group, which had a 96.67% survivorship. Pupal exposure to 800 ppmv for 20 min was sufficient to completely inhibit adult emergence. Larvae exhibited greater tolerance to ozone gas generated by cold plasma than eggs and pupae 24 h after exposure. Additionally, ozone treatment at 400 and 800 ppmv significantly increased total hemolymph protein content after 24 h, reaching 17.55 g/L and 18.50 g/L, respectively, compared to 16.52 g/L for the control. Cold plasma ozone treatment altered the stored beeswax matrix by modifying essential hydrocarbons and enhancing fatty acid diversity, while preserving the fundamental ester structure, thus converting it into a functionally changed biomaterial. Exposure to ozone gas at 800ppmv caused remarkable abnormalities in appearance in wax moths throughout their life stages, resulting in shrunken, twisted, and crippled insects unable to complete normal development.

**Supplementary Information:**

The online version contains supplementary material available at 10.1038/s41598-026-36802-w.

## Introduction

The greater wax moth, *G. mellonella* (Lepidoptera: Pyralidae), poses a substantial threat to honeybee colonies, mainly due to the destructive feeding behaviors of its larvae. These larvae burrow into the honeycomb, creating silk-lined tunnels that lead to a condition called galleriasis. This disorder can be harmful for emerging bees, as they may become trapped and face starvation^1–3^. The larvae can rapidly colonize a hive, causing extensive damage within just a week^1^. Their presence obstructs bee movement and feeding, as they create dense webs and holes in the cell caps, leading to honey leakage^4^. Such infestations can severely impact bee populations, with weaker colonies potentially abandoning their hives and stronger ones facing complete destruction, which poses economic challenges for beekeepers^5^.

Traditionally, controlling *G. mellonella* has relied on chemical pesticides and fumigants. However, these means can harm non-target organisms, including beneficial insects like honeybees^6–10^. While chemical fumigants such as phosphine have been thoroughly studied, concerns regarding their toxicity and potential residues in food have limited their use^11,12^. Moreover, the repeated application of these chemicals has led to resistance among pests, requiring higher doses for effective control^13,14^.

Given the growing demand for safe pest control methods and pesticide-free food options, researchers are seeking alternatives to manage wax moth populations without harming bees or disrupting the ecosystem^15–17^. Non-chemical and non-thermal methods have appeared as promising solutions for pest management. These methods include microwave heating, infrared heating, ozone treatment, ultraviolet light, and cold plasma technology^12^. Cold plasma operates at temperatures below 40 °C and has demonstrated significant potential as a physical disinfection method, thanks to its user-friendly nature, cost-effectiveness, and safety profile^18,19^.

An innovative approach to pest control is the use of ozone (O_3_) fumigants generated through cold plasma technology. This method involves applying high-voltage electric discharge to oxygen, which generates ozone—a highly reactive gas known for its strong oxidizing properties^20,21^. One of the key advantages of ozone is its ability to break down into harmless oxygen within 20 to 50 min at room temperature, making it a safer alternative for various applications. The United States Environmental Protection Agency (USEPA) recognizes ozone as “Generally Recognized As Safe” (GRAS), and the Food and Drug Administration (FDA) has approved its use for food and water purification^22^, and reducing aflatoxin contamination^23,24^.

In addition to its broad applications in disinfection, sterilization, preservation, and fumigation^25^, it effectively targets pests while minimizing harm to beneficial organisms^26^. This feature allows beekeepers to integrate ozone treatment into their existing hive management practices as a non-chemical alternative^27^.

Research has extensively explored the effectiveness of gaseous ozone in eliminating insects across various commodities, employing different application techniques in both laboratory and field conditions^28–33^. Our study was conducted focusing on the effects of cold plasma-generated ozone on various life stages of *G. mellonella*. The research examined changes in larval hemolymph protein profiles and evaluated the impact of ozone on wax comb composition, as well as the occurrence of malformations in larvae, pupae, and adult moths. These investigations aim to shed light on the potential of using cold plasma-generated ozone as an effective non-chemical pest control strategy for managing *G. mellonella*.

## Materials and methods

### Ozone gas production

**Fig. 1:**
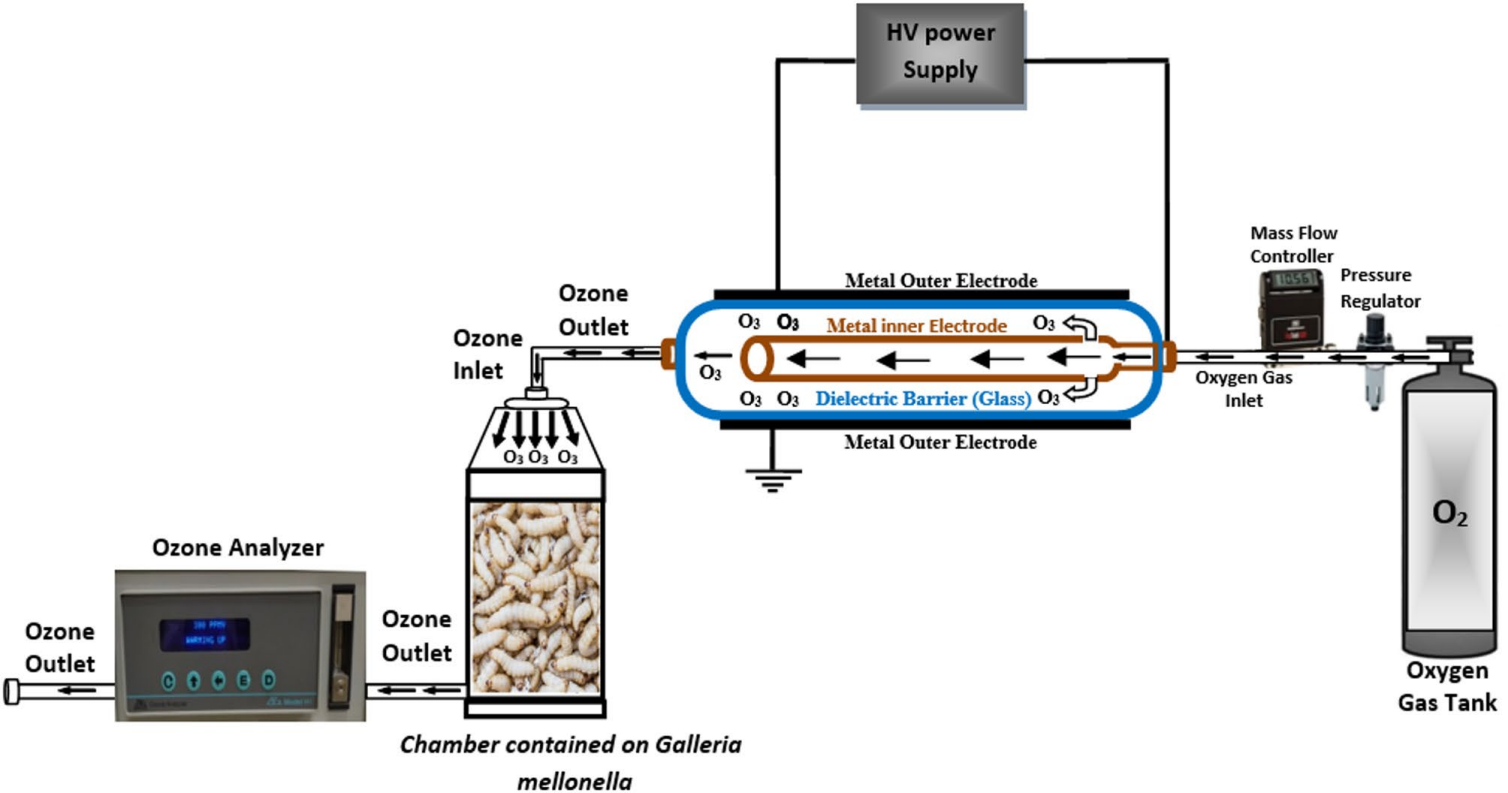
Schematic diagram of the ozone generation and fumigation setup. The figure was created using Microsoft Office (Version 2010; Microsoft Corporation, Redmond, WA, USA; https://www.microsoft.com by inserting and combining several shapes to illustrate the concept.

Ozone gas generated by a coaxial atmospheric pressure (DBD) reactor. This non-thermal plasma system was developed at the Department of Physics, Faculty of Science, Al-Azhar University. During the production process oxygen gas was introduced. A discharge voltage applied to two coaxial electrodes separated by a glass dielectric creates a filament discharge. Electrons generated in this discharge have sufficient energy to break down oxygen molecules, forming ozone (O_3_). The DBD cell was filled with oxygen gas at a flow rate of 0.5 L/min, and the discharge current was adjusted to regulate the created ozone concentration produced^34^. *G. mellonella* eggs, larvae and pupae were directly placed into jars and exposed to ozone treatment. The exposure fumigation chamber continuously replenished ozone gas to avoid its half-life of 20–30 min during the insect exposure period. The consistent flow of ozone gas through the fumigation chamber ensured that the concentration of ozone inside the jars remained stable. An ozone analyzer (Model H1-AFX Instrumentation, USA- San Diego, California, United States of America) was used to measure the ozone concentration within the jars. The AC input voltage was 220 V at 50 Hz, and a voltage transformer connected the two outer and inner electrodes separated by a gap, as shown in (Fig. 1).

### Characterization of electrical properties

**Fig. 2:**
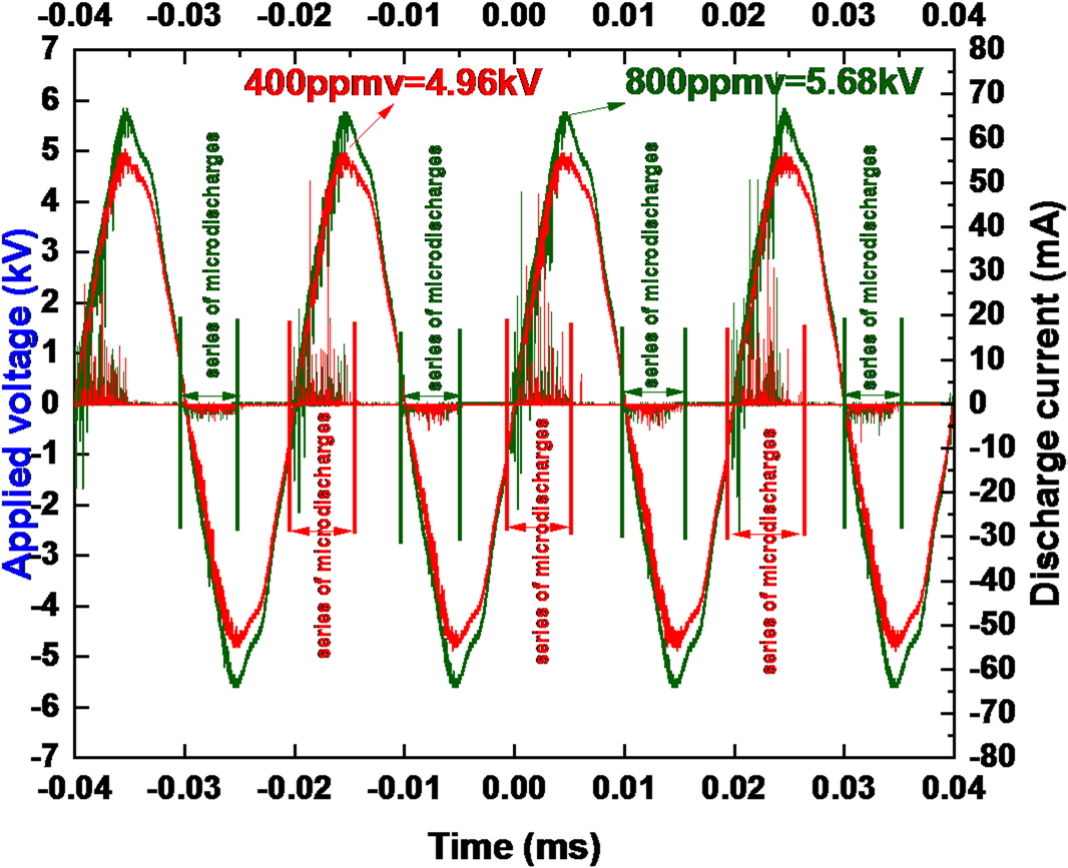
Schematic picture of the feeding voltage V(t) and discharge current I(t).

The oscillogram of the current-voltage relationship in the coaxial DBD reactor system was captured at room temperature and atmospheric pressure, as depicted in (Fig. 2). When the applied voltage exceeds the breakdown voltage in the DBD, filamentary mode discharge current occurs. Waveforms of the current and voltage supplied to the coaxial DBD reactor at an oxygen flow rate of 0.5 L per minute for ozone generation show different ozone concentrations of 400 and 800 parts per million by volume (ppmv) at voltage levels of 4.96 kV and 5.68 kV, respectively (Table 1).

**Table 1:** Values of applied voltage and corresponding power at different Ozone concentrations.

Ozone concentration	Applied voltage (kV)	Electric power *P*_el_(Watts)
400ppmv	4.96 kV	84 W
800ppmv	5.68 kV	270 W

**Table 2:** Estimated the expected exposure times (min) to achieve 50% and 95% mortality in *G. mellonella* eggs, larvae, and pupae following 24 h exposure to cold plasma-generated Ozone treatment.

Stage		LT_50_ (min)			LT_95_(min)			Slope ± SE
		Value	Confidence limits		Value	Confidence limits		
			Lower	Upper		Lower	Upper	
Eggs	400	3.10	1.6	4.59	76.26	54.2	126.59	1.2 ± 0.14
Larvae		26.12	13.3	49.2	152.9	174.1	960.4	2.1 ± 0.16
Pupae		13.20	6.9	20.3	78.94	67.5	266.3	2.1 ± 0.16
Eggs	800	2.02	0.99	3.13	24.14	18.61	34.78	1.5 ± 0.21
Larvae		20.41	12.8	30.2	103.4	84.7	254.4	2.3 ± 0.16
Pupae		2.2	0.9	3.59	70.6	47.1	136.5	1.08 ± 0.16

### Power measurement method

**Fig. 3:**
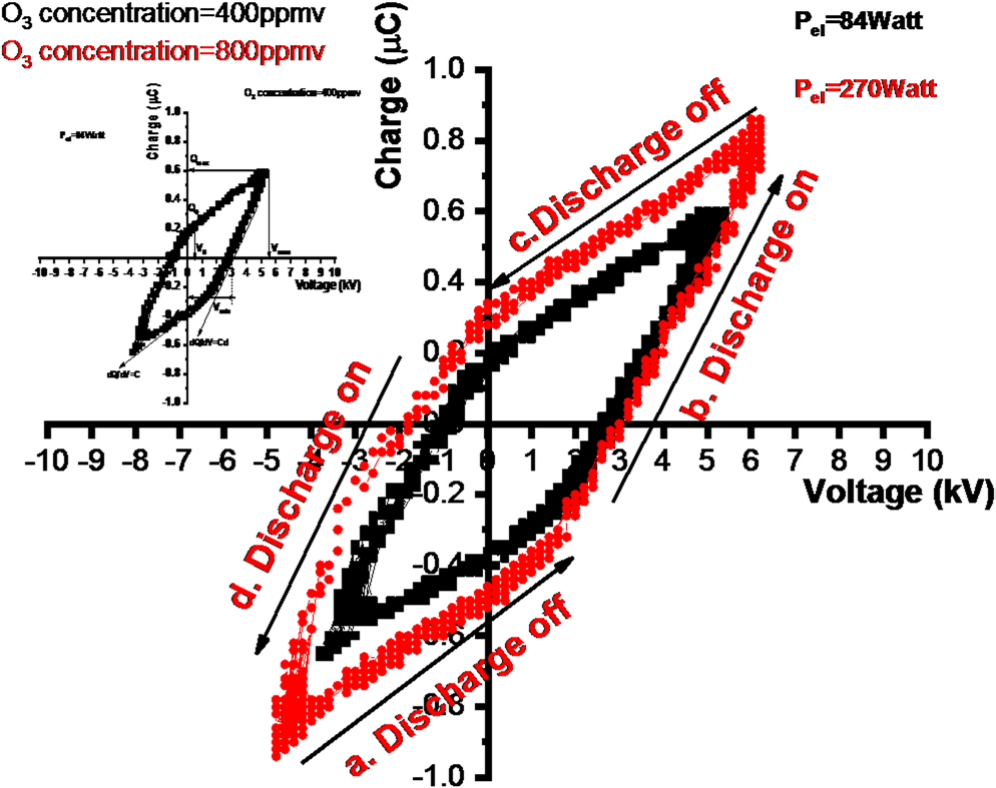
Lissajous diagrams measured for ozone at flow rate of 0.5 (l/min).

The analysis of the power was performed according to the original work of Manley (1943), who utilized the voltage-charge Lissajous figures to characterize average consumed power through discharge^35,36^. The charge–voltage characteristic plot is illustrated in (Fig. 3). The capacitance of the effective discharge value was indicated by obtaining two distinct slopes of the Q–V plot.

### Insect culture

In the warmer months of March, April, and May, we collected larger wax moths larvae from contaminated storage combs at the Plant Protection Research Institute’s Bee Research Department. Once we had identified the contaminated combs, we sent specimens to the Plant Protection Research Institute’s Taxonomy Department to confirm that the larvae were larger wax moths. After this confirmation, we carefully transported the infested combs to the laboratory at the Stored Products Department Plant Protection Research Institute, Doki, Giza, Egypt, for further analysis.

The laboratory population of *G. mellonella* was reared in glass jars on artificial diet according to a previously described protocol^37^. The jars were covered with muslin cloth and secured with rubber bands at a constant temperature of 28 ± 2 °C with a relative humidity of 45 ± 5% to ensure access to all stages of the insect. Freshly laid eggs were collected daily using a fine camel-hair brush and transferred to sterile Petri dishes lined with moist filter paper to maintain humidity. To ensure egg viability, they were examined under a stereomicroscope. Only healthy, intact eggs less than 24 h old were selected for ozone exposure. Larvae were observed daily for feeding activity, moulting, and signs of microbial contamination. The last larval instars were selected for ozone exposure according to the head capsule width. Pupation was observed after approximately 18–21 days. Pupae were carefully separated and transferred to clean containers for ozone exposure.

### Susceptibility of different life stages of *G. mellonella* to ozone gas generated by cold plasma

The study investigated the susceptibility of different life stages of the greater wax moth (*G. mellonella*) to ozone gas generated by cold plasma. Three life stages were tested: eggs, larvae, and pupae. Specimens were collected and stored in sterile glass containers before being exposed to ozone gas at concentrations of 400 or 800 ppmv for varying durations (0, 5, 10, 20, 40, 60, or 80 min) under controlled conditions of 28 ± 2 °C and 45 ± 5% relative humidity.

For the egg stage, approximately 150–200 eggs (24–72 h old) were collected after adult moths laid them on wax paper. After ozone exposure, the eggs were monitored daily for hatching, pupation, and adult emergence until no further emergence was noted for two days. Unhatched eggs were considered dead.

For the larvae stage, 20 larvae of the last larval instar were placed in sterile containers, and mortality was evaluated after 24 h. The percentage of pupation and adult emergence was monitored similarly to the eggs.

For the pupae stage, 20 pupae were also placed in sterile containers sealed with nylon window screens. They were monitored for adult emergence after treatment, with incubation lasting up to 24 h for larvae and 4 weeks for pupae under the same temperature and humidity conditions.

Control groups for each life stage were maintained under identical conditions without ozone treatment. The experiments were repeated three times with three replications for each stage, including control groups.

### Effects of ozone gas generated by cold plasma on hemolymph protein level profiles of *G. mellonella* larvae

#### Collection of hemolymphs

Hemolymph was collected from the last larval instar of *G. mellonella* by puncturing the larval abdomen with a fine needle. A few crystals of phenylthiourea (PTU) were added to prevent melanization. Hemolymph was centrifuged at 10,000 x g for 10 min at 4 °C to remove hemocytes and cell debris, and the supernatant was kept in a freezer at −20 °C for analysis. The assay of total protein content of the larval hemolymph from the challenged larvae and control larvae was assayed according to Bradford’s method^38^.

#### Electrophoresis of hemolymph samples

Electrophoresis of cell-free hemolymph was performed using a 15% SDS polyacrylamide gel (SDS-PAGE)^39^. The hemolymph from each control and treated larva was dissolved in 5X sample buffer with 2% (w/v) 2-mercaptoethanol and then heated for 5 min at 95 °C. Denatured proteins from each hemolymph sample were loaded into designated wells of the stacking gel, and a standard protein marker ranging from 5 to 245 kDa was included. The samples were then covered with electrode buffer, and electrophoresis was run in vertical slab gel units. After electrophoresis, the gels were stained using Coomassie Brilliant Blue R250 stain (50% dH_2_O, 40% methanol, 10% glacial acetic acid, and 0.1% Coomassie Brilliant Blue) for 20 min with gentle agitation. Subsequently, they were destained overnight in a destaining solution (50% dH_2_O, 40% methanol, and 10% glacial acetic acid)^40^. The analyses of the gels were performed using ImageJ Analyzing Software^41^.

#### Effects of ozone gas generated by cold plasma on wax

To determine the effect of ozone gas generated by cold plasma on beeswax composition, three strips of wax base (6 × 12 cm) were placed in a fumigation chamber and exposed to 800 ppmv ozone gas generated by cold plasma for 2 h. The hydrocarbons extracted from each sample and standard were analyzed according to a previously described protocol^42^. The fatty acids extracted from each sample and standard were converted to the corresponding methyl esters using the necessary diazomethane solution^43^. Peak identification and total area determination were performed according to a previously described protocol^43–45^. Ester values ​​were determined according to previously described protocol^46^ and calculated as indicated.

#### Observation of insect morphology

The jars that contain larvae were observed regularly until the emergence of the adults to assess inhibition of larvae, pupae and adult morphological malformations. The examination was carried out at the center laboratory in Plant Protection Research Institute, Doki, Giza, Egypt. A stereomicroscope 20MP was used to examine the morphology of *G. mellonella* at various life stages. The digital images were taken with a ToupCam (Ver. 3.7) 18 MP digital camera.

### Statistical analysis

Data were analyzed by using procs ANOVA and REG in SAS^47^. For each of the tested life stages, percent mortality and factorial analysis were used in ANOVA. Ozone gas exposure time and concentration were the main effects, and mortality was the dependent variable.

To achieve the best fit for the obtained results, both exposure times and tested concentrations were transformed into logarithmic values using the formula log₁₀ (value + 1). Accordingly, the statistical model was expressed as:

$${\mathrm{Y}} = {\mathrm{a}} \pm {\mathrm{log}}_{{10}} \left( {{\mathrm{Exposure}}\;{\mathrm{time}}} \right) \pm {\mathrm{log}}_{{10}} \left( {{\mathrm{Power}}} \right).$$

multiple regression models (determination of the significant effect of multiple factors on one or more aspects) were used. R-square values presented the goodness of fitting, while the P values presented the significant probability of the tested factors (i.e., exposure times and tested concentrations)^48^.

The reactor (coaxial dielectric barrier discharge) power formula is shown in (Eq. 1)^49^, where the total power (P_el_) is regarded as the operating frequency (ƒ) and the peak voltage V_max_. The Lissajous figure shows the minimum external voltage V_min_ at which ignition occurs, and the electric energy consumed per voltage cycle (Eel) and the electric power (Pel) canbe estimated by the following relations^50^.

1 $$\begin{aligned} Eel & = 2\left( {Vmax~Q0~ - ~Qmax~V0} \right) \equiv {\mathrm{Area}}\;{\mathrm{of}}\left( {Q~ - ~V} \right)~\;{\mathrm{diagram}} \\ Pel & = \frac{{Eel}}{T} = fEel \\ \end{aligned}$$

## Results

**Fig. 4:**
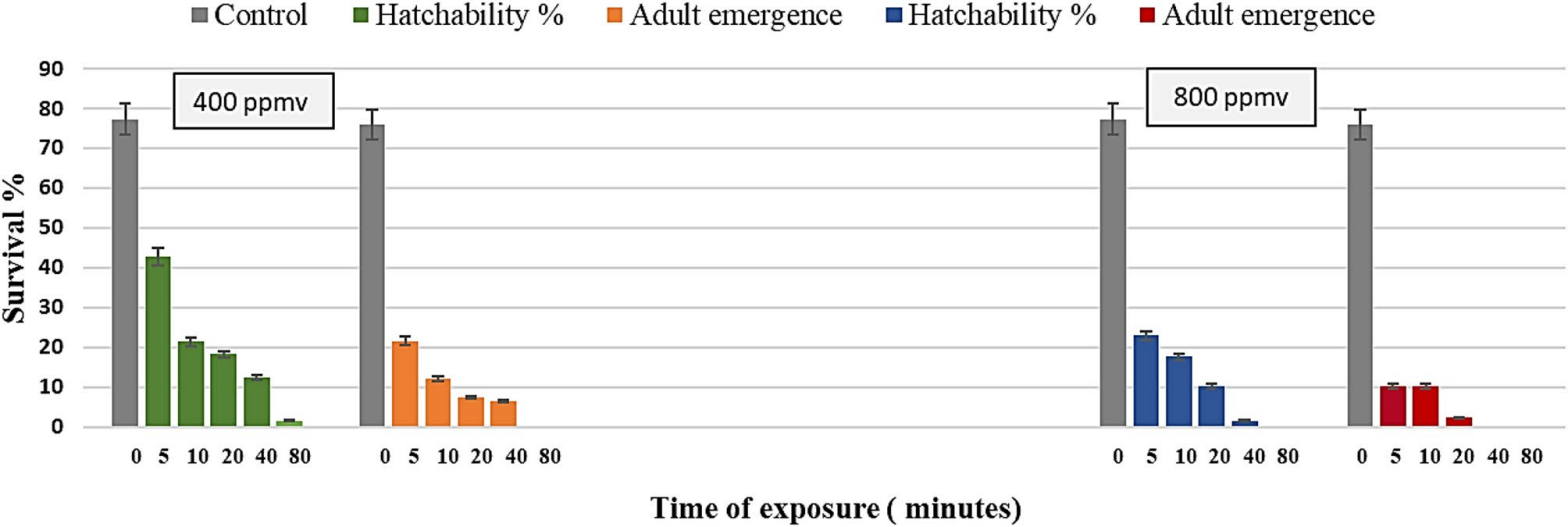
Impact of ozone gas on *G. mellonella* egg stage after different exposure times at 400 and 800 ppmv.

The impact of ozone gas generated by cold plasma at 400 and 800 ppmv on the egg stage of *G. mellonella* was profound, as detailed in (Fig. 4). A significant reduction in the number of larvae, pupation, and adult emergence percentage was observed (*P* < 0.01) with increased exposure duration to both 400 and 800 ppmv ozone gas. Notably, exposure of eggs to 800 ppmv of ozone gas for 40 min resulted in complete prevention of both pupation and adult emergence, underscoring the sensitivity of the egg stage to ozone exposure.

**Fig. 5:**
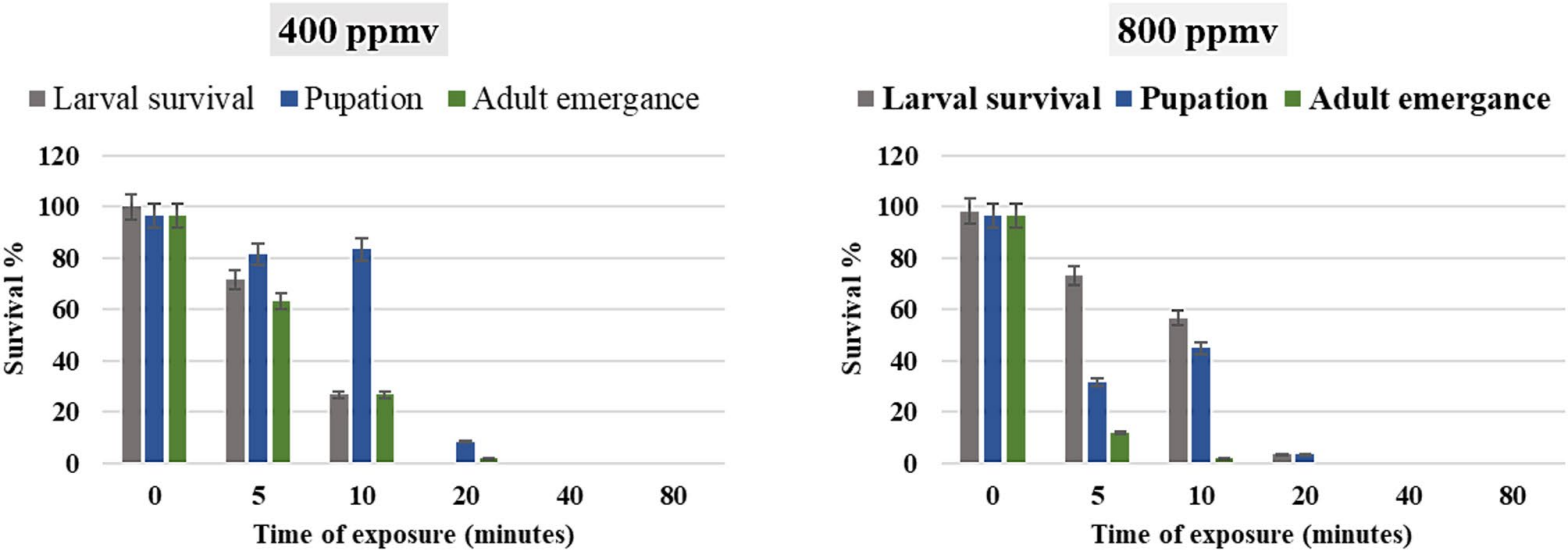
Impact of ozone gas on *G. mellonella* larval stage after different exposure times at 400 and 800 ppmv.

The effects of ozone gas generated by cold plasma at 400 and 800 ppmv on the larval survival, pupation percentage and adult emergence of *G. mellonella* resulting from larval treatment were significant and dose-dependent (Fig. 5). Mortality increased with exposure time (*P* < 0.01) at both 400 ppmv and 800 ppmv concentrations. For example, at 400 ppmv, exposure for 40 min resulted in 100% mortality after 7 days, while exposure for 80 min achieved the same result in just 1 day. At the higher concentration of 800 ppmv, effects were more rapid, with 20 min of exposure leading to 100% mortality after 7 days, and those subjected to 80 min reached the same mortality percentage within 1 day. The effects were more rapid at the higher concentration, with a significant reduction in both pupation and adult emergence noted. Specifically, exposure of larvae for 20 min resulted in complete suppression of larval instar survival after 40 min and 0.0% pupation after 20 min, compared to the control group. Overall, these results corroborate the findings across both concentrations, demonstrating a significant increase in mortality percentage with prolonged exposure times (*P* < 0.01).

**Fig. 6:**
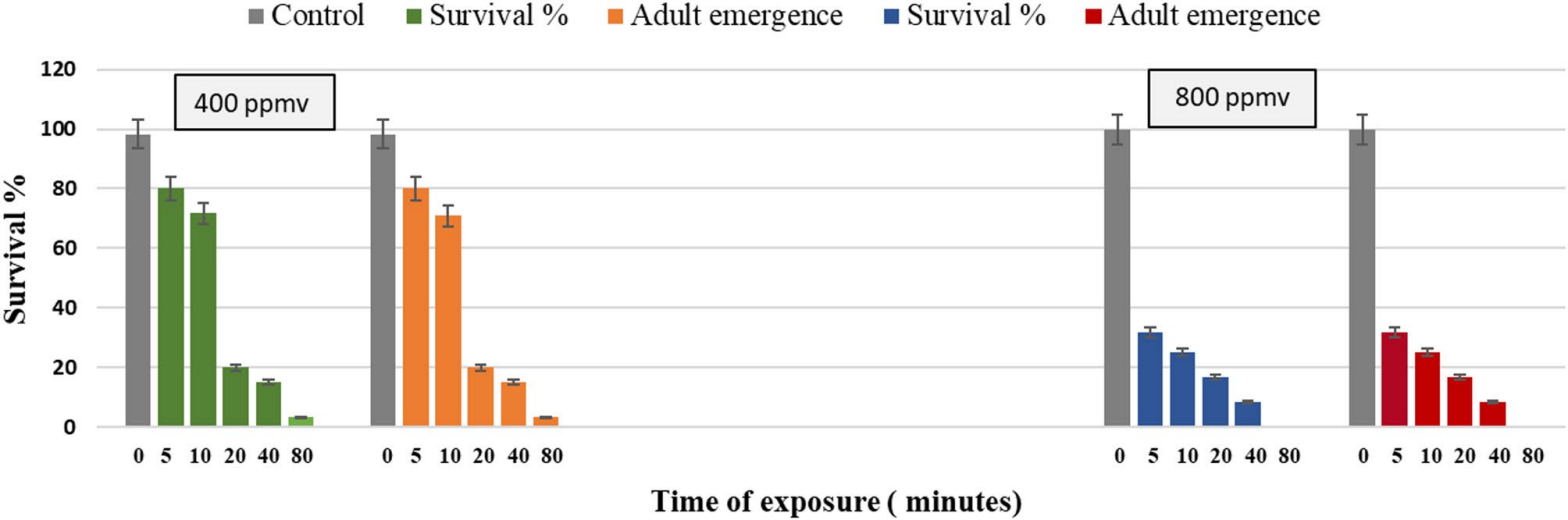
Impact of ozone gas on *G. mellonella* pupal stage after different exposure times at 400 and 800 ppmv.

The effects of ozone gas generated by cold plasma on the mortality percentage and adult survivorship from the treated pupal stage of *G. mellonella* post exposure to 400 and 800 ppmv of ozone gas generated by cold plasma at various time periods were analyzed and are summarized in (Fig. 6). A significant decrease in the number of newly emerged adults was observed with increasing exposure time and concentration of ozone gas. The adult emergence rates ranged from 80.0 to 3.33% and 31.67 to 0.0% for 400 and 800 ppmv, respectively, compared to the control groups, which exhibited emergence rates of 98.33 and 100%, respectively. Notably, pupae exposed to 800 ppmv of ozone gas for 60 min experienced complete inhibition of adult emergence, with 100% mortality recorded in this group.

The estimated exposure durations required to achieve 50% (LT₅₀) and 90% (LT₉₀) across different developmental stages after 24 h post-exposure to two concentrations (400 and 800 ppmv) of ozone gas generated by cold plasma were illustrated in (Table 2). The data indicated a decrease in expected exposure time with increasing ozone concentration for all stages. Larvae demonstrated a higher tolerance to ozone gas, requiring longer exposure times (26.12 min at 400 ppmv and 20.41 min at 800 ppmv) to achieve 50% mortality compared to eggs and pupae, which were significantly more sensitive, requiring only 3.10 min and 2.02 min at 400 ppmv and 13.20 min and 2.2 min at 800 ppmv, respectively.

Determination of the significant effect of exposure time and tested concentration factors on the mortality percentage of immature stages of *G. mellonella* after 24 h post-exposure to ozone gas is presented in (Table 3). The duration of the insects’ exposure to the ozone gas turns out to plays a much more significant role in determining their survival than the concentration of the gas itself (*P* < 0.0001). Strong relationships were found for all three life stages—eggs, larvae, and pupae—indicating that longer exposure durations result in higher mortality rates. The relationship is relatively strong for eggs (R2 = 0.3647), stronger for larvae (R2 = 0.5266), and even stronger for pupae (R2 = 0.6328), according to the findings. On the other hand, varying the concentration did not have a significant impact on the mortality of eggs and larvae, (*P* = 0.58 and 1.00) and (R² = 0.3647 and 0.5266).

**Table 3:** Determination of the significant effect of exposure times and concentrations on the mortality percent of *G. mellonella* egg, larvae and pupae following 24 h exposure to cold plasma-generated Ozone treatment.

Multiple regression parameter	Egg		Larvae		pupae	
	Slope	*P*	Slope	*P*	Slope	*P*
Log Exp Time slope	−0.54	0.0001	−0.933	0.0001	−0.976	0.0001
Log Concentration slope	−0.009	0.58	−1.77	1.00	−0.040	0.0256
R^2^	0.3647		0.5266		0.6328	

Determinations of the significant effect of exposure time, tested pest stages and power level factors on adult emergence percentage of *G. mellonella* following exposure to ozone gas are summarized in (Table 4). Ozone gas significantly decreased adult emergence rates of *G. mellonella* across all tested factors, (*P* < 0.05). The best results were seen with higher ozone concentrations (800 ppm) and longer exposure durations (60–80 min), as emergence rates decreased to 1.22 and 0.56%, respectively, compared with 90.61% of controls. Pupae showed the greatest resistance, with an emergence rate of 34.05%, while eggs were the most vulnerable, with only 15.92% successfully emerging.

**Table 4:** Determinations of significant-effect of exposure-times, tested-pests stage and concentrations on adult emergence% of *G. mellonella* following 24 h exposure to cold plasma-generated Ozone treatment.

Factor	Level	Mean ± SE	Factor	Level	Mean ± SE
Concentration	400	28.841 ± 3.12 ^a^	Time	0	90.61 ± 2.58^a^
				5	45.02 ± 6.3 ^b^
	800	22.25 ± 2.89 ^b^		10	28.78 ± 5.17 ^c^
				20	7.74 ± 2.00 ^d^
F		9. 85		40	4.96 ± 1.44 ^d^
P		0.002		60	1.22 ± 0.64 ^d^
LSD		4.161			
Stage	Egg	15.92 ± 3.95 ^a^		80	0.56 ± 0.56 ^d^
	Larvae	26.667 ± 5.64 ^b^	F		141.97
	Pupae	34.048 ± 5.61^c^	P		0.0001
F		25.1	LSD (0.05)		7.7845
P		0.0001			
LSD (0.05)		5.0962			

Means, in the same column, followed by the same letter are not significantly different using the LSD test at *P* < 0.05.

### Protein analysis in larval hemolymph

The investigation into protein levels in the larval hemolymph of *G. mellonella* following 24 h of ozone exposure at 400 and 800 ppmv is summarized in (Table 5). A two-way ANOVA revealed a significant increase in total protein concentrations post-exposure. The protein profiles, outlined in (Table 6) (Supp. Fig. S1) demonstrated peptide molecular weights ranging from 35 to 97 kDa. Notably, the intensity of peptide bands at 97 and 90 kDa increased significantly after ozone exposure, and a new peptide band around 58 kDa emerged, indicating the presence of inducible peptides. Interestingly, a peptide at 81 kDa was present in the hemolymph of larvae treated with 400 ppmv and in the control sample but was absent in those treated with 800 ppmv, suggesting a concentration-dependent effect on protein expression.

**Table 5:** Changes in total haemolymph protein of *G. mellonella* last larval instar following 24 h exposure to cold plasma-generated Ozone treatment.

Sample	Total protein content (g/L)			Mean±	STD	SE	
Control	16.3	16.64	16.61	16.52 ± 0.1^c^	0.188	0.061	
400ppm		17.4	17.63	17.61	17.55 ± 0.07^b^	0.127	0.042
800ppm	18.4	18.56	18.55	18.50 ± 0.05^a^	0.090	0.029	

SE: standard error. STD: standard deviation. Different letters represent significant differences among means (*p* < 0.05).

**Table 6:** Molecular weight (KDa) and relative fragmentation (Rf) of haemolymph protein pattern for challenged and healthy samples of last larval instar of *G. mellonella*.

Parameters			1	2	3	poly morphism
Band No.	Rf	Mw	Cont.	Sample challenged with 400ppm of ozone	Sample challenged with 800ppm of ozone	
1	0.31	97	+	+	+	Monomorphic
2	0.35	90	+	+	+	Monomorphic
3	0.37	83	+	+	+	Monomorphic
4	0.39	81	+	+	-	Polymorphic
5	0.42	75	+	+	+	Monomorphic
6	0.44	71	+	+	+	Monomorphic
7	0.48	58	-	+	+	Polymorphic
8	0.65	42	+	+	+	Monomorphic
9	0.72	36	+	+	+	Monomorphic
10	0.73	35	+	+	+	Monomorphic
Total			9	10	9	

### Analysis of hydrocarbons, fatty acids and ester in beeswax following treatment to ozone gas generated by cold plasma

#### Targeted degradation and reconfiguration of hydrocarbons

The analysis of hydrocarbons in beeswax treated with ozone gas generated by a cold plasma from a coaxial atmospheric pressure (DBD) reactor presented in (Table 7). Ozone treatment resulted in a clear and specific rearrangement of the beeswax’s hydrocarbon profile. The focused, near-complete degradation of particular alkanes and the creation of new compounds were two opposing processes, even though there was a 15.3% net rise in total hydrocarbons. The most notable alterations were the substantial declines in C36 (−94.7%) and C34 (−76.9%). On the other hand, most other hydrocarbons, including the main constituent C30, showed a steady and mild decrease of roughly − 13.3%. New hydrocarbons C21, C31, and C38 were formed; these were not present in the control.

**Table 7:** Determination of major hydrocarbons and change% in beeswax following cold plasma-generated Ozone treatment.

Compound	Type	Untreated (Area%)	Treated (Area%)	Change%
C11	BB	23.63%	20.49%	−13.3%
C13	BB	1.63%	1.42%	−12.9%
C16	VV	1.93%	1.67%	−13.5%
C17	VV	1.80%	1.56%	−13.3%
C21	VV	Not Detected	0.86%	New
C27	BV	1.05%	0.91%	−13.3%
C29	VV	1.50%	1.30%	−13.3%
C30	VB	9.57%	8.29%	−13.4%
C31	BV	Not Detected	0.40%	New
C33	BV	15.57%	13.51%	−13.2%
C34	VB	1.78%	0.41%	−76.9%
C35	BV	4.96%	4.30%	−13.3%
C36	VB	9.49%	0.50%	−94.7%
C38	VV	Not Detected	0.42%	New
C40	VV	2.65%	2.30%	−13.2%
Total Hydrocarbons		4034	4652	+ 15.3%

Note: Hydrocarbon type is indicated by structure codes: ***B*** = Branching methyl group, ***V*** = Straight-chain methylene group. Codes represent the bond environment (e.g., VB indicates a bond between a straight chain and a branch).

#### Profound oxidation and enrichment of the fatty acid profile

The analysis of fatty acid in beeswax treated with ozone gas generated by a cold plasma from a coaxial atmospheric pressure (DBD) reactor presented in (Table 8). The fatty acid fraction had the most dramatic change. We found that the net amount of total fatty acids increased by 51.4%. Alongside this, there was a significant redistribution of molecules. While some acids, like caprylic acid, had a significant increase (+ 191.7%), others, like undecanoic acid, were almost completely eradicated (−99.8%). Crucially, new polyunsaturated fatty acids (PUFAs) such as gamma-linolenic and linoleic acid were formed in the treated wax but not in the control. Additionally, the long-chain omega-3 PUFA eicosapentaenoic acid (EPA) was dramatically enriched by 212%.

**Table 8:** Determination of change % in the fatty acid profile of beeswax following cold plasma-generated Ozone treatment.

Fatty acid	Untreated (Area%)	Treated (area%)	Change%
Butyric acid	3.76%	2.87%	−23.7%
Caproic acid	2.83%	1.01%	−64.3%
Caprylic acid	0.72%	2.10%	+ 191.7%
Enanthic acid	2.51%	0.42%	−83.3%
Undecanoic acid	6.43%	0.01%	−99.8%
Lauric acid	Not detected	0.01%	New
Myristic acid	0.57%	0.17%	−70.2%
cis-10-pentadecanoic	Not detected	0.13%	New
Palmitic acid	Not detected	0.29%	New
Palmitoleic acid	Not detected	0.54%	New
Gamma-Linolenic acid	Not detected	0.82%	New
Eicosapentaenoic acid	3.56%	11.10%	+ 212%
Behenic acid	2.16%	2.05%	−5.1%
Erucic acid	0.68%	Not detected	Eliminated
Total fatty acids	317.17	480.24	+ 51.4%

#### Preservation of the core wax ester structure

The ester values and change% in beeswax treated with ozone gas generated by a cold plasma from a coaxial atmospheric pressure (DBD) reactor presented in (Table 9). The beeswax’s fundamental structural integrity was maintained. A statistically insignificant change was seen in the ester value, a crucial measure of the integrity of the high-molecular-weight wax esters, from 345.02 mg KOH/g in the untreated wax to 342.21 mg KOH/g in the treated wax (a drop of only 0.81%).

**Table 9:** Determmination of beeswax ester value and change% following cold plasma-generated Ozone treatment.

Sample	Ester Value (mg KOH/g)	Change (%)
Untreated	345.02 ± 0.67	-
Treated	342.21 ± 0.65	−0.81%

#### Observation of insect morphology

**Fig. 7:**
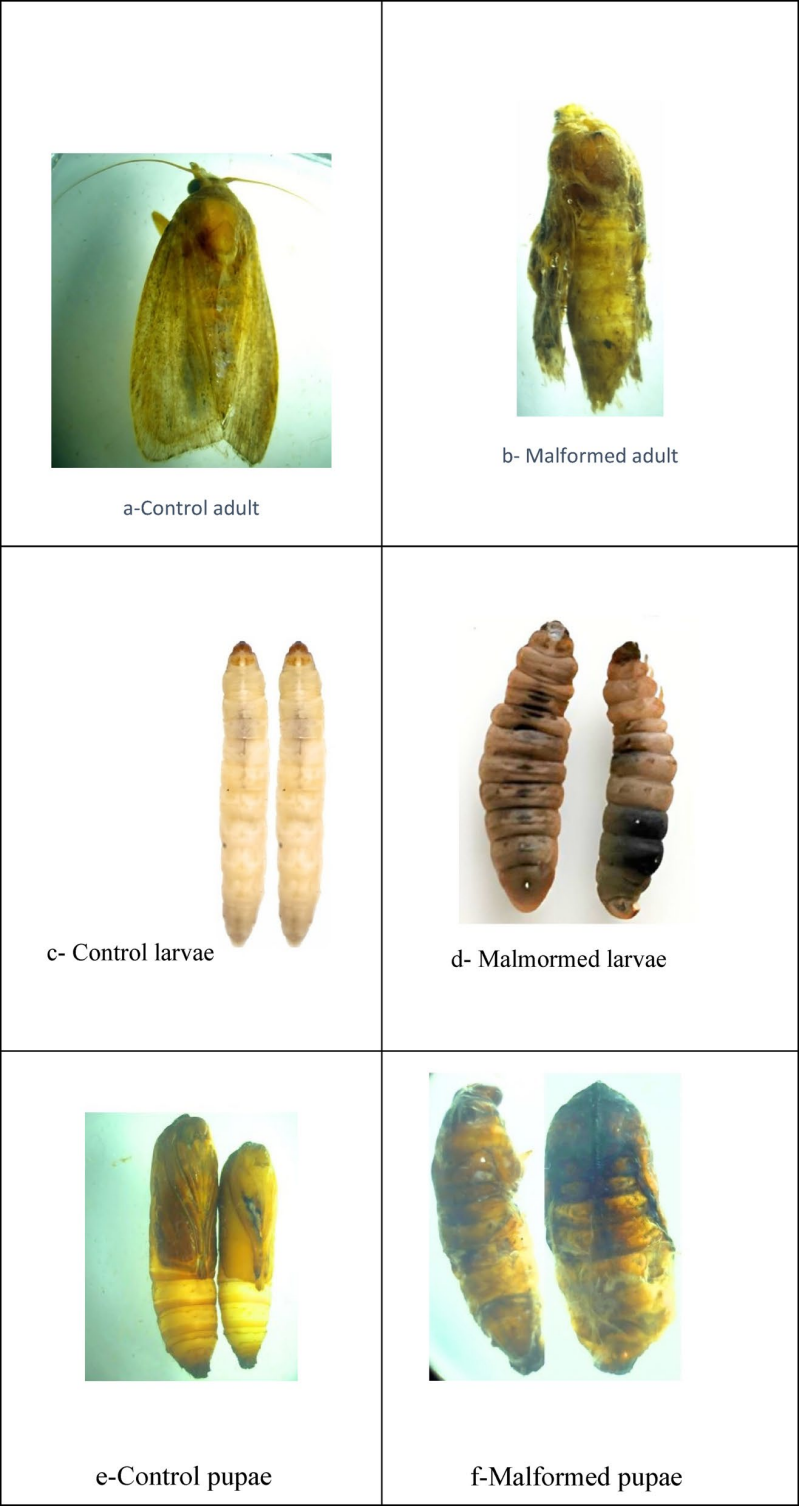
Morphological abnormalities in *Galleria mellonella* life stages (x10 magnification) following exposure to 800 ppmv ozone gas. (**a**, **c**, **e**) Control insects showing normal development. (**b**, **d**, **f**) Ozone-treated insects displaying severe malformations. (**a**, **b**) Adult; (**c**, **d**) Larval; (**e**, **f**) Pupal.

*G. mellonella* exposure to 800 ppmv ozone gas resulted in marked morphological abnormalities across all development stages as compared to the untreated control group. Control larvae, pupae, and adults had normal coloration, smooth cuticles, and well-formed body segmentation (Fig. 7a, c, and e). In contrast, adult moths emerging from ozone-treated groups showed inadequate wing extension, torn or folded wings, and cuticular distortion (Fig. 7b). ozone-treated larvae had darker integuments, shrunken and twisted bodies, and irregular segmentation (Fig. 7d). Pupal abnormalities were also present, including irregular sclerotization, shriveled bodies, and blackish discoloration (Fig. 7f).

## Discussion

In this study, we found that ozone gas exposure duration and concentration a significant affected the mortality of *G. mellonella* larvae, pupae, and eggs. Higher ozone concentrations (800 ppmv) for 40 and 20 min eliminated larval survival without any pupation. This is consistent with prior research showing increased mortality in *Plodia interpunctella* larvae exposed to 50 ppmv of ozone over three days, and an 86.3%^28^.

Furthermore, our observations are consistent with studies demonstrating the susceptibility of various insect stages to ozone gas. Mortality rate in *Tribolium confusum* larvae due to ozone exposure. Moreover, complete mortality in *Ephestia kuehniella* larvae occurred after six days at 1 g/m³ for 5 h, while *Oryzaephilus surinamensis* and adult *P. interpunctella* experienced complete mortality after shorter exposures of 6 h at 30 ppmv and 60 min at 500 ppmv, respectively^51–54^. Additionally, *E. cautella* larvae had a mortality rate of 38.92% after 1 h with cold plasma ozone exposure, reaching complete mortality after 5 h^55^.

Our findings demonstrate that the combined effects of UV photons and ozone in cold plasma negatively affected the emergence of *G. mellonella* eggs. In other studies, UV radiation also harmed *T. castaneum* eggs, causing surface tissue damage^56^; however, in this study, 100% mortality was achieved within 80 min using 800 ppmv of ozone gas. Previous studies have shown that ozone is effective in managing a variety of insect pests^57–61^.

Our findings further suggest that the embryonic stages of *G. mellonella* are especially susceptible to ozone gas, supporting previous studies showing that ozone affects tissue differentiation and cell division, resulting in mortality and decreased egg hatching rates^62^. Even at low concentrations, ozone may affect these stages by destroying soluble cuticular proteins and allowing reactive oxygen species to enter through the outer membrane of the egg^63,64^. Additionally, as earlier research has indicated, the disruption of the eggshell’s cell membranes due to an accumulation of charges that exceed its surface strength^65^. Ozone-induced anoxic or hypoxic conditions may elevate the respiration rate and decrease the metabolic processes of *G. mellonella* eggs, leading to delayed or absent hatching. Additionally, it can impact the embryo’s nervous system, leading to retardation and blocking of egg hatching^66^.

It was observed that the time required to achieve 50% and 90% mortality decreased with increasing exposure duration and ozone concentration across all developmental stages following 24 h of ozone gas treatment. According to the study’s findings, better control of *G. mellonella’s* immature stages results in a shorter time (LT_50_ and LT_90_) needed for mortality, which in turn leads to the shortest time required for mortality. Furthermore, following a 24-hour exposure to ozone gas, time was found to be a significant determinant of death in immature stages, supporting results from previous research that highlights the significance of exposure length in predicting lethality. Time was the main factor impacting death in immature stages after exposure to ozone gas for 24 h^19^.

Our findings demonstrate that *G. mellonella* eggs exhibited more sensitivity to ozone gas at concentrations of 800 and 400 ppmv. This aligns with previous research that noted similar sensitivities in other insect species under varying conditions. The eggs of *P. interpunctella* were significantly affected by exposure to ozone gas concentrations of 3 and 5 ppmv for 120 min^67^.

In biological research, *G. mellonella* has been extensively utilised as a model organism for studying insect immunological responses^68,69^. Various studies have documented differing responses based on the type of challenge presented. Specifically, as a response to these challenges, insects release proteins into the hemolymph, reflecting their specialization and adaptation in the organism. Proteins serve crucial roles in insect biology, acting as antibacterial agents^70^ detoxifying agents^71^, hormone carriers^72^, morphogenesis proteins^73^ or even exhibiting similarities to certain human proteins.

Our investigation revealed a significant increase in the total protein content of *G. mellonella* larvae hemolymph after 24 h of exposure to ozone gas at concentrations of 400 and 800 ppmv, compared to the control group. Numerous authors documented the electrophoretic protein bands and total protein alterations following different pest treatments^74–76^. This increase suggests a concentration-dependent effect consistent with findings from studies on *Spodoptera mauritia* larvae^77^. Similarly, elevated protein content has been documented in other insect species, including the cotton leafworm *Spodoptera littoralis*, when exposed to specific chitin synthesis inhibitors^78^. Conversely, decreased total protein levels were observed in 5th instar larvae of *Pericallia ricini* treated with *Azadirachta indica* leaf extract compared to control larvae^79^.

In the current study, the SDS-PAGE analysis revealed alterations in the protein profiles of *G. mellonella* larvae hemolymph following 24 h of exposure to different concentrations of ozone gas. Specifically, there was an increase in the intensity of the 97 and 90 kDa bands after exposure to 400 and 800 ppmv concentrations of ozone gas compared to the control group. This observation suggests that the impact of ozone gas on these hemolymph proteins is dependent on the concentration used, which may be a potential influence on protein synthesis or degradation. Previous research has shown that ionizing radiation affects the protein profile of *S. littoralis* hemolymph using gel electrophoresis^80^. These studies revealed that gamma radiation influenced the cellular immune system and protein expression, potentially leading to insect death due to disturbances in the immune function and protein integrity. furthermore, gamma radiation has been reported to have variable effect on the number and intensity of the protein bands in *Sarcophaga* bullata^81^.

In our investigation, we identified the emergence of a new band with a molecular weight of 58 kDa following treatment with both 400 and 800 ppmv concentrations of ozone gas, while this band was absent in the control group. This result supports findings from previous studies that suggest the appearance of new bands may be attributed to the generation of free radicals that directly impact nitrogenous compounds, leading to peptide linkage breakdown and fragmentation of protein molecules^82^. The observed changes in protein levels may reflect a dynamic balance among the processes of synthesis, storage, transport, and degradation of structural and functional nutrients, as well as physiological responses to specific exogenous substances^81^. Moreover, several factors are related to the mechanism through which ozone generated by cold plasma induces insect mortality. It initiates stress through reactive oxygen or nitrogen species and modifies DNA via plasma components like high-energy UV photons. Free radicals can impact antioxidant enzymes such as lipid peroxide, catalase, and glutathione reductase, inducing oxidative stress^19^. Additionally, charged particles cause surface damage. Within the insect’s body, oxidation of lipids, amino acids, and nucleic acids occurs, ultimately leading to insect death^61,66,67^.

According to the study’s findings, the oxidative processes seen throughout the studies are responsible for the notable changes in the hydrocarbon profile of beeswax. In particular, we observed rise in the overall amount of detectable hydrocarbons, which suggests the emergence of novel volatile pieces. This finding is in line with other researchers’ hypotheses, which contend that oxidative activities cause both the creation of new chemical species and the breakdown of preexisting ones. Furthermore, the formation of new hydrocarbons shows that oxidation destroys preexisting molecules while also promoting molecular breakage and recombination. These results highlight the intricacy of the chemical changes that take place in beeswax during oxidative stress^82,83^.

In this investigation, we saw notable changes in the fatty acid composition. The concept of oxidative breakdown of more complex structures is supported by the 51.4% net increase in total fatty acids, which indicates a significant release or synthesis of lipid components. The results from other investigations that suggest some fatty acids may be selectively broken down while others are produced or enriched are consistent with this molecular redistribution^84^. The fact that some acids are almost eliminated while others are significantly increased emphasizes how dynamic these changes are. Crucially, in line with earlier studies, the appearance of novel polyunsaturated fatty acids (PUFAs) in the treated wax that were not present in the control provides a definite sign of lipid peroxidation mechanisms in action^85^. The stability of this parameter confirms that the ozone treatment does not destroy the fundamental character of the wax but rather modifies its accessory components, proving that it selectively targets the more reactive hydrocarbon and free/fragmented lipid components without causing significant hydrolysis of the stable ester bonds that form the primary scaffold of the beeswax^86^.

This indicates that ozone treatment enhances the qualities of beeswax while preserving its essential properties. Moreover, ozone gas treatment does not affect the ester value of beeswax, indicating that ester compounds are preserved. Furthermore, an 800 ppmv ozone gas concentration had no discernible effect on the foundation’s colour, flexibility, or comb quality. These results, which are consistent with earlier research showing the preservation of important chemical components in organic materials exposed to ozone treatment, add support to the possible application of ozone treatment for decontaminating beeswax in apiculture. Ozone can lower pesticide residues, but it may also momentarily change the sensory properties of beeswax^87^. Ozone technology is a non-thermal food preservation technique that increases food safety without sacrificing quality or posing a threat to the environment^88^.

Beeswax’s main chemical constituents, esters, hydrocarbons, and fatty acids, incur substantial deterioration and change when exposed to the oxidative conditions produced by ozone from cold plasma. By producing reactive oxygen species (ROS), such as ozone, cold plasma produces a very reactive environment that targets the chemical makeup of the wax^89^.

In the current study, the observed abnormalities in *G. mellonella* following ozone exposure can be reveals a consistent pattern of oxidative and developmental disruption across the life stages of this pest, which most likely degraded the insect’s cellular structures and cuticular proteins. Ozone is a powerful oxidizing agent that can degrade lipids, proteins, and hydrocarbons in the insect cuticle, diminishing flexibility and structural integrity during molting and adult emergence. Other insects, such as *Agrotis ipsilon*, have shown similar effects, with ozone exposure inducing oxidative stress and physiological alterations, resulting in lower growth and developmental problems. These can harm cellular membranes, proteins, and DNA, resulting in diminished growth, developmental abnormalities, or death^90^. Furthermore, *A. ipsilon* pupae exposed to varying ozone concentrations had deformities particularly during critical transformation stages^91^.

## Conclusion

Honeybee colonies face significant threats from the wax moth *G. mellonella*, which has traditionally been managed with chemical fumigants that can compromise honey quality. This research investigates the use of cold plasma-generated ozone gas as a safer and more efficient alternative for pest control. Exposure to *G. mellonella* at concentrations of 400 and 800 ppmv resulted in increased mortality rates across different life stages, effectively suppressing development. Ozone exposure also significantly elevated the overall hemolymph protein concentration in honeybees. Additionally, the treatment altered the beeswax matrix, potentially benefiting honeybee health. This study highlights the potential of ozone gas in promoting sustainable apiculture. Further research into its broader applications will enhance our understanding of its long-term impacts on honeybee colonies. Investigating honey quality will ensure that there are no adverse effects on the final product. Comparisons with alternative approaches to pest control.

## Supplementary Information

Below is the link to the electronic supplementary material.

Supplementary Material 1

## Data Availability

Data will be made available on request by contacting with Abeer O. Abotaleb.
