# Supplementary material for: Effects of cold plasma generated ozone on development of Galleria mellonella induced alterations in hemolymph protein and biochemistry of beeswax
Source: Sci Rep. 2026 Feb 10;16:5935. doi: 10.1038/s41598-026-36802-w (PMC12894833; doi:10.1038/s41598-026-36802-w)
Supplement: Supplementary file 1 — Supplementary Material 1 [file 41598_2026_36802_MOESM1_ESM.pdf]

Supplementary materials

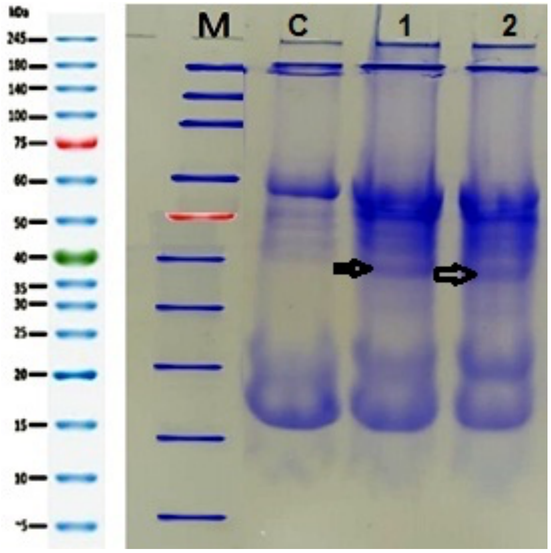

Fig. Supp. S1- Protein profiles of larvae hemolymph of *G. mellonella* of both control (lane C) and treated ones with 800 ppmv of ozone (lane 1), 400ppmv (lane 2), protein marker (lane M).
